# Long-term Survival Rate and Clinical Quality of Individually Layered Indirect Composite Restorations in Adolescents and Young Adults

**DOI:** 10.3290/j.jad.b5825410

**Published:** 2024-11-13

**Authors:** Britta Hahn, Alina-Kathrin Holst, Annette Ilse, Imme Haubitz, Karl Halbleib, Norbert Krämer, Gabriel Krastl, Sebastian Soliman

**Affiliations:** a Dentist, Department of Conservative Dentistry and Periodontology, University Hospital of Würzburg, Germany. Wrote the manuscript, performed treatments in study center 1, supervised clinical examinations and SEM analysis.; b Dentist, Department of Conservative Dentistry and Periodontology, University Hospital of Würzburg, Germany. Performed clinical examinations and SEM analysis for a doctoral degree.; c Private dental practice for Paediatric and Adolescent Dentistry, Frankfurter Str. 32, 65830 Kriftel, Germany. Performed treatments in study center 2.; d Biometrician, Department of Conservative Dentistry and Periodontology, University Hospital of Würzburg, Germany. Consulted on and performed statistical evaluation.; e Dental Technician, Department of Conservative Dentistry and Periodontology, University Hospital of Würzburg, Germany. Manufactured all restorations.; f Head of the Department of Pediatric Dentistry, University Hospital Gießen, Department of Paediatric Dentistry, University Hospital of Gießen, Germany. Contributed substantially to the protocol and the manuscript.; g Chairman, Department of Conservative Dentistry and Periodontology, University Hospital Würzburg, Germany. Contributed substantially to the manuscript, performed treatments in study center 1.; h Dentist, Department of Conservative Dentistry and Periodontology, University Hospital of Würzburg, Germany. Idea, performed treatments in study center 1, contributed substantially to the manuscript.

**Keywords:** adolescent dentition, adult dentition, clinical quality parameters, indirect composite restorations, qualitative margin analysis, survival

## Abstract

**Purpose:**

To evaluate the survival and clinical quality of individually layered indirect composite restorations (ICRs) in the mixed and permanent dentition at two study centers.

**Materials and Methods:**

A total of 155 adhesively cemented ICRs in 34 participants (aged 6 to 50 years and treated between 2008 and 2018) were evaluated for survival and clinical quality. All were individually layered restorations fabricated from laboratory sculptable composites by a specialized dental technician. Two calibrated independent investigators examined and graded each restoration as success, survival with repair, or failure based on the FDI criteria. The marginal quality and gap width of the restorations were analyzed by scanning electron microscopy. The periodontal health of treated teeth (TT) was evaluated in comparison with that of unrestored control teeth (CT) by measuring the pocket depth (PD), clinical attachment level (CAL), sulcus bleeding index (SBI), and the modified Turesky Plaque Index (TPI). A serial t-test (p <0.05) was used for statistical analysis of periodontal parameters. Success and functional survival rates were calculated using the Kaplan–Meier method.

**Results:**

Molar incisor hypomineralization (MIH) was the most common indication for treatment (41%). The median age at treatment was 14.9 years (68%-CI: 7.7–29.5). The median service time of the restorations was 5.7 ± 3.4 years. 132 restorations were classified as a success, 21 as survival with repair, and 1 as a failure. The success rates at 1, 5, and 10 years were 95.4%, 87.4%, and 78.8%, respectively, and the corresponding functional survival rates were 100.0%, 98.9%, and 98.9%. The clinical quality, encompassing esthetic, functional, and biological criteria, was rated as excellent or good in over 90%. Periodontal response, however, was the only criterion showing worse results since restored teeth (TPI = 1.9) had significantly more plaque than CT (TPI = 1.7; p = 0.0001). No significant differences were observed in PD, CAL, or SBI. The mean marginal gap width was 135.7 µm and 63.8% of the restorations had perfect margins.

**Conclusion:**

ICRs are suitable for minimally invasive restoration of large tooth structure defects in the developing dentition of children and adolescents and for long-term temporary restoration of the adult dentition.

The demand for indirect composite restorations (ICRs) has increased significantly in recent years.^54^ These restorations can be fabricated manually with sculptable light-curing composites or by using the CAD/CAM fabrication process. Although their short-term success and survival rates are slightly lower than those of silicate ceramics, some studies have reported comparable rates.^8,19,39^ While conclusive data on the longevity of ICRs are still lacking, the longevity of ceramic restorations can be considered clinically proven.^49^

All-ceramic restorations are more wear- and fracture-resistant^1,6,63^ and less prone to plaque retention than ICRs.^11^ Long-term studies have shown that the most common cause of failure is secondary caries for ICRs and fracture for indirect ceramic restorations.^57^ Compared to ceramic restorations, ICRs offer the advantage of having a greater fatigue resistance and higher load-bearing capacity despite a comparable or thinner minimum layer thickness.^40,41,52^ This allows optimal preservation of dental hard tissue even under unfavorable conditions such as limited space.^20,26,55,59,60^ Some sculptable indirect composite resins are preheated in an oven to reduce their residual monomer content and enhance their conversion rate and physical properties.^46^ To increase the fracture strength of both individually layered and CAD/CAM composite restorations even further, glass fibers can be integrated.^43,50,51^ Due to their reduced resistance to abrasion,^15^ ICRs do not interfere with the proper eruption and position of young teeth. In addition, composites can be integrated into orthodontic treatment regimens with relative ease, and composite surfaces are more conducive to adhesive bonding of orthodontic elements than ceramic surfaces.^7,28^ This suggests that ICRs may be indicated in significantly younger patients than indirect ceramic restorations. This assumption is based on the results of a clinical study^35^ involving a young female who underwent full-mouth rehabilitation with adhesively cemented all-ceramic restorations for amelogenesis imperfecta at the age of 12. Given the patient’s young age, the authors attempted to minimize the amount of tooth preparation and maintain the recommended minimum occlusal layer thickness. However, subsequent grinding measures likely resulted in violation of the minimum layer thickness as this patient had the highest number of fractured ceramic restorations. Nevertheless, they reported survival rates of 99% after 5 years and 91% after 10 years for all-ceramic restorations in patients with non-carious defects.

In adults, permanent all-ceramic restorations may also be contraindicated or associated with risks in certain cases, such as bite raising. When planning to increase the vertical dimension of occlusion (VDO), composite restorations must be used as long-term temporaries to test the compatibility of the planned occlusal changes over an extended period of time, ie, for diagnostic provisionalization.^24^ Indirect composite restorations are a useful minimally invasive or non-invasive long-term temporary solution in these situations.^15,24^

However, indirect sculptable composites also have disadvantages, such as the inclusion of small air bubbles during layering, a lower wear resistance, a higher residual monomer content, and increased water absorption compared to restorations fabricated with industrially polymerized CAD/CAM materials.^2,3,44,45,47^

To date, the majority of data on restorations made with sculptable laboratory composite materials are from short-term observational studies, and only two studies have reported long-term outcomes 5 and 10 years after restoration placement, respectively.^5,20,21,27^ These include a clinical follow-up study of laboratory-fabricated ICRs in children and adolescents with non-carious tooth structure defects due to conditions such as molar incisor hypomineralization (MIH), amelogenesis imperfecta, and dentinogenesis imperfecta, which showed that all 34 ICRs placed in a total of eight young patients aged 6 to 15 years were still functional after observation periods ranging from 2 months to 2 years.^20^ The fabrication process for laboratory-made ICRs is quite complex and requires a high level of dental technology and expertise, which may explain the lack of available data and low rates of use of these restorations.

For severely worn or decayed deciduous teeth, prefabricated stainless-steel crowns have been used as a cost-effective and well-accepted treatment option,^58^ also for restoring adolescent dentition.^10,62^ They are reported to have a high survival rate (95% at 3 years).^62^ Their disadvantages are poor esthetics and poorer marginal fit compared to individually made crowns.^36^

The objective of this two-center observational study was to evaluate the quality and longevity of individually layered, ICRs in teeth with non-caries-related structural defects (including MIH) over a 10-year observation period. The research hypothesis was that the survival rate of ICRs is not lower than those of all-ceramic restorations and prefabricated stainless-steel crowns mentioned above.^35,62^

## Materials and Methods

### Treatment Regimen

**Fig 1:**
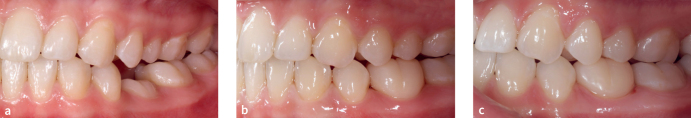
Non-invasive restoration of persistent primary tooth 75 in an adolescent female. *(a)* Initial situation following fixed orthodontic treatment. *(b)* Situation immediately after restoration. *(c)* Situation at 3.5-year follow-up.

**Fig 2:**
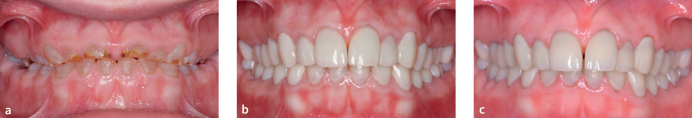
Minimally invasive full-mouth rehabilitation in an adolescent female with dentinogenesis imperfecta. *(a)* Initial situation. *(b)* Situation after bite raising and restoration with ICRs. *(c)* Situation at 5.5-year follow-up.

**Fig 3:**
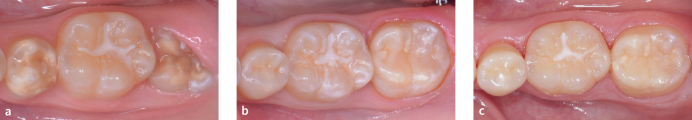
Adolescent male with molar incisor hypomineralization (MIH). *(a)* Initial situation with view of tooth 35, tooth 37 with partial MIH before completion of eruption. *(b)* Situation after restoration of tooth 35 with a partial composite crown and of tooth 37 with a composite resin inlay. *(c)* Situation at 4.5-year follow-up.

**Fig 4:**
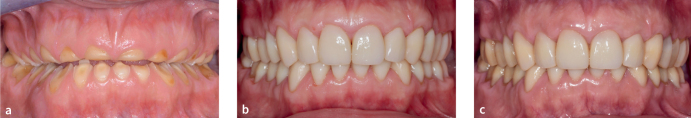
Diagnostic provisionalization with long-term temporary ICRs for bite raising in a 31-year-old male with erosive tooth structure loss. *(a)* Initial situation. *(b)* Situation after bite elevation with ICRs. *(c)* Situation at 3.5-year follow-up.

The indications for the ICRs in the mixed and permanent dentition can be divided into four indication groups (Figs 1–4):

- Persistent primary teeth with infraocclusion
- Congenital structural anomalies
  - *Amelogenesis imperfecta, dentinogenesis imperfecta*
- Acquired structural anomalies
  - *Molar incisor hypomineralization (MIH)*
- Long-term temporary restoration
  - *Diagnostic provisionalization in VDO elevation due to erosion or abrasion, for example*

All participants included in this study had one of these indications and were treated by clinicians specializing in restorative dentistry (n = 12 operators in study center 1; n = 1 operator in study center 2). All preparations had a rounded shoulder margin design. In cases of MIH tooth preparation was extended into sound hard tissue. In cases of persistent primary teeth with infraocclusion, dental erosion, hypomaturated amelogenesis imperfecta and dentinogenesis imperfecta tooth preparation was limited to smoothing the occlusal relief to preserve as much enamel as possible. All restorations were airborne-particle abraded (50 µm alumina, 1.5 bar) and luted using either light-curing flowable composite (Tetric Evo Flow, Ivoclar Vivadent, Schaan, Liechtenstein) if the maximum layer thickness of the restoration was ≤1.5 mm or dual-curing composite cement (Multilink, Ivoclar Vivadent, Schaan, Liechtenstein) if the restoration layer thickness was greater than 1.5 mm.

All restorations were fabricated with microfilled composite (SR Adoro, Ivoclar Vivadent; dimethacrylate: 17–19 wt%, filler content: 64–65 wt%, silica particle size 10–100 nm)^34^ individually layered using a variety of dentin, effect and enamel materials, and resin shades in the dental laboratory by a dental technician with specialized training in this type of restoration. When technically feasible, the restorations were additionally reinforced with glass fibers (Stick Net, GC Europe, Leuven, Belgium), which were placed evenly on the occlusal cavity floor and subsequently infiltrated with a liner (SR Adoro Liner, Ivoclar Vivadent).

### Study Design and Participants

**Fig 5:**
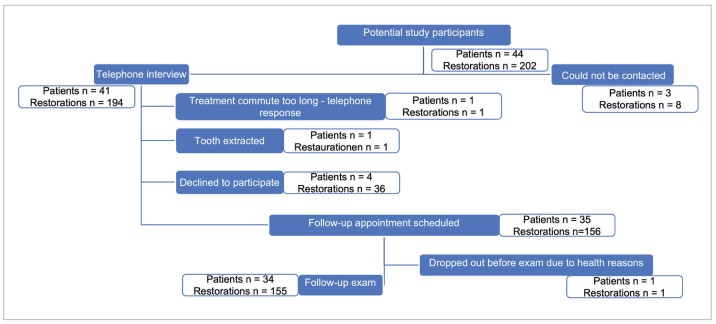
Flow chart of the inclusion and exclusion process, including the number of patients and restored teeth per subgroup.

The study protocol was approved by the local ethics committee (reference number: 38/19-me) on August 26, 2019. All subjects who received ICRs at the study centers (center 1: Department of Restorative Dentistry and Periodontology, University Hospital Würzburg, Germany; center 2: private dental practice for pediatric and adolescent dentistry, Dr. A. Ilse, Germany) between 2008 and 2018 and were aged between 6 and 50 years at the time of restoration, were eligible for recruitment. A total of 44 eligible subjects with 203 ICR-treated teeth were identified by retrospective medical records review, and the authors attempted to contact each one by phone or mail. Of the 44 eligible subjects, three could not be contacted, four refused to participate without giving a reason, two could only be interviewed by phone because they had either moved out of the area (n = 1) or had lost the affected tooth (n = 1). The remaining 35 were scheduled for a study visit, but one dropped out prematurely due to hospitalization. The remaining 34 participants (study group) gave their written informed consent, and their 155 ICRs were clinically evaluated (n = 25 inlays, n = 43 partial crowns, n = 87 crowns). The flow chart in Figure 5 summarizes the inclusion and exclusion process and indicates the number of participants and ICRs in each subgroup.

### Clinical Examination

Study visits for all 34 participants (study center 1: n = 25, study center 2: n = 9) were scheduled between 10/2019 and 10/2020. The standardized visit consisted of (1) a description of the study aims and procedures and obtaining informed consent; (2) patient history and documentation of treatment indication; (3) intraoral examination; (4) assessment of periodontal parameters; (5) professional tooth cleaning and oral hygiene instruction; (6) clinical quality grading of the ICRs according to the FDI criteria^30^; (7) standardized intraoral photographic documentation (Canon EOS 80D, SIGMA ring flash, TAMRON 90 mm macro lens); and (8) preparation of a partial silicone impression (Honigum, DMG, Hamburg, Germany) for later assessment of marginal quality by scanning electron microscopy (SEM). In subjects with multiple ICRs, the restoration to be analyzed was arbitrarily selected. A retraction cord (Ultrapak CleanCut, Ultradent Products, South Jordan, UT, USA) was used to facilitate visualization of subgingival margins.

The clinical outcome of each restoration was evaluated by two calibrated examiners equipped with a conventional diagnostic light and diagnostic probes based on the following periodontal parameters: (1) pocket probing depth (PPD) and (2) clinical attachment level (CAL), (3) sulcus bleeding index (SBI), and (4) Turesky’s modification of the Quigley–Hein plaque index (TPI). PPD and CAL measurements were obtained using a manual periodontal probe (#CP-12, Hu-Friedy; Chicago, IL, USA). Each periodontal parameter was recorded at six sites per treated tooth (TT) and control tooth (CT). A control tooth was defined as the first untreated tooth distal to the treated tooth in the same quadrant. If no distal tooth was available, the mesial tooth served as the control tooth. In full-mouth rehabilitation, no control tooth was available.

### Outcome Definition

Failure (F) was defined as total decementation and/or irreparable damage to the restoration. Success (S) was defined as retention of the intact restoration in situ without failure. Survival with repair (SR) was defined as a less damaging event such as chipping, secondary caries, decementation, and/or the need for interproximal shape correction. In the case of SR, the reason for failure and the type of repair performed were documented in the patient records.

### SEM Analysis

**Fig 6:**
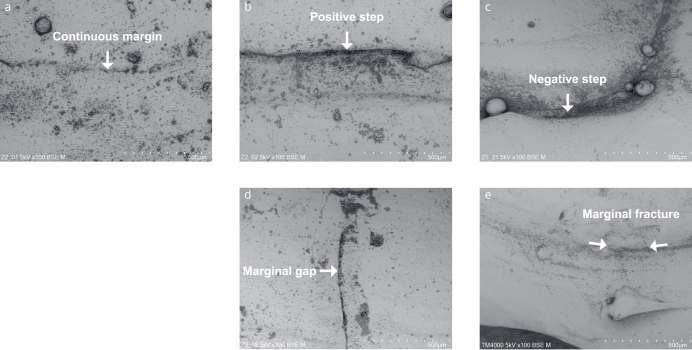
Representative SEM images of the five marginal quality outcomes: *(a)* continuous margin, *(b)* positive step, *(c)* negative step, *(d)* marginal gap, and *(e)* marginal fracture.

For SEM analysis of the buccal/vestibular and oral/lingual/palatal sites, one randomly selected restoration per subject (n = 29) was replicated with epoxy resin (RenCast CW 2215/Ren HY 5162, Gößl Pfaff, Karlskron, Germany). The epoxy resin was mixed according to the manufacturer’s instructions and poured into the silicone impressions mentioned earlier. After 24 h of storage in an incubator (Memmert, Schwabach, Germany) at 37°C, these cured replicas were sputter-coated with gold in a sputter coater (EMITECH K550 Emitech, Taunusstein, Germany). Marginal gap width was measured as the distance between the restoration margin and the cavity margin. Marginal quality was assessed based on the percentage of continuous margins, positive steps, negative steps, marginal gaps, and marginal fractures using a tabletop scanning electron microscope (Hitachi TM4000Plus) at 100× to 1,000× magnification. Results for each quality outcome variable were expressed as a percentage of total margin length. Examples of the five marginal quality outcomes are shown in Figure 6a–e.

### Statistical Analysis

For descriptive analysis of the continuous variables, the data set was characterized by the number of non-missing values, mean, standard deviation, median, 68%-confidence interval (68% CI), minimum, and maximum values.

The overall and functional survival rate of the indirect composite resin restorations, expressed as 1-, 5-, and 10-year survival rates, were calculated using the Kaplan–Meier method. Overall survival includes all restorations that did not receive or were not in need of repair. Functional survival includes all restorations that incurred an event requiring repair during their time of service.

The differences in the periodontal parameters PD, CAL, SBI, and TPI between the test and CT were evaluated using a serial t-test, considering the intra-individual correlation. A probit model with Lilliefors limits was used to test the data for Gaussian normal distribution. The significance level was set at α = 0.05.

## Results

### General Data

Thirty-four of 44 eligible participants treated at the two study centers from 2008 to 2018 were included in this retrospective study (Fig 2), yielding an overall recall rate of 77% (34/44). At the time of restorative treatment, the 34 participants (20 female, 14 male) had a mean age of 18.7 ± 12.8 years (median 14.9 years). They were followed for a mean of 5.7 ± 3.4 years (min 1.4 years, max 11.8 years).

Table 1 shows the composition of the study population by treatment indication and age. MIH was the most common indication for ICRs. Indication groups I to III consisted of adolescents, and group IV consisted of adults.

**Table 1:** Mean age of study subjects (n = 34) at the time of treatment by indication group (I–IV)

	Indication	n	%	Age at treatment	
				Mean	SD
I	Persistent primary teeth/infraocclusion	10	29.41	18.15	4.86
II	Congenital structural anomalies: Amelogenesis imperfecta, dentinogenesis imperfecta	4	11.76	14.48	7.02
III	Acquired structural anomalies: Molar incisor hypomineralization	14	41.18	9.78	3.11
IV	Long-term temporary restoration Due to erosion, abrasion	6	17.65	42.97	6.68

Of the 155 ICRs studied, 114 (73.5%) were cemented with dual-cure composites and 41 (26.5%) were cemented with flowable light-cure-only composites.

### Survival Analysis

**Fig 7:**
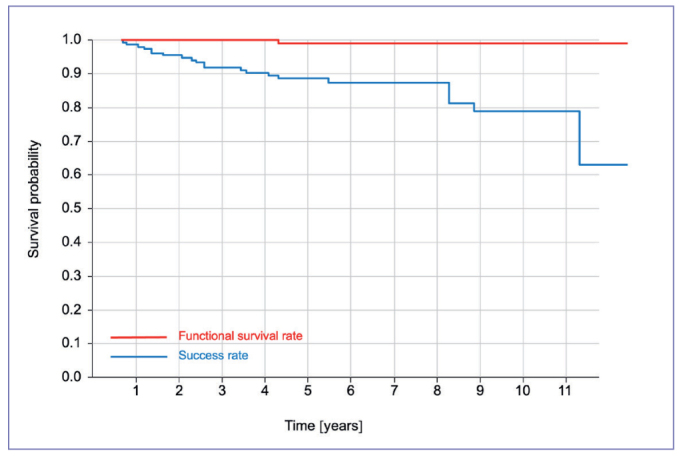
Kaplan–Meier graphs of functional survival and success for all restorations (n = 155). The functional survival rate was 100.0% after 1 year, 98.9% after 5 years, and 98.9% after 10 years. The success rate was 95.4% after 1 year, 87.4% after 5 years, and 78.8% after 10 years.

The ICRs were observed for an average of 5.7 years. The results of the Kaplan–Meier analysis of success (S) and functional survival (FS) of the ICRs are presented in Figure 7. The K-M estimates for success (S) and FS at 1, 5, and 10 years were 95.4%, 87.4%, and 78.8% (S) and 100%, 98.9%, and 98.9% (FS), respectively.

### Failure Analysis

**Fig 8:**
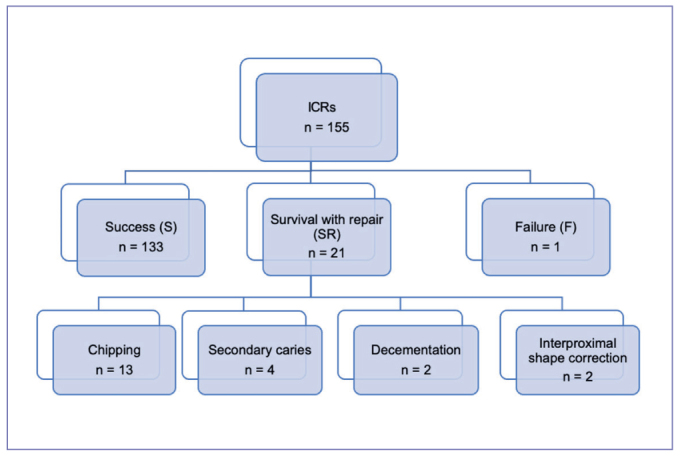
Detailed failure analysis of the 155 ICRs evaluated in this study.

Figure 8 shows the relative failure rates and causes of failure. Of the 155 ICRs studied, 133 were classified as success (S), 21 as survival with repair (SR), and one as failure (F). During the follow-up period, 22 ICRs (SR + F) developed problems. Twenty-one were minor failures that could be repaired; these restorations remained in place and were classified as SR. The most common cause of failure was chipping (n = 13), followed by secondary caries (n = 4), decementation (n = 2), and interproximal shape correction (n = 2). One restoration was a total failure (F) due to secondary caries and was lost to follow-up. Clinical examples of success, survival with repair, and failure are shown in Figure 9a–c.

**Fig 9a:**
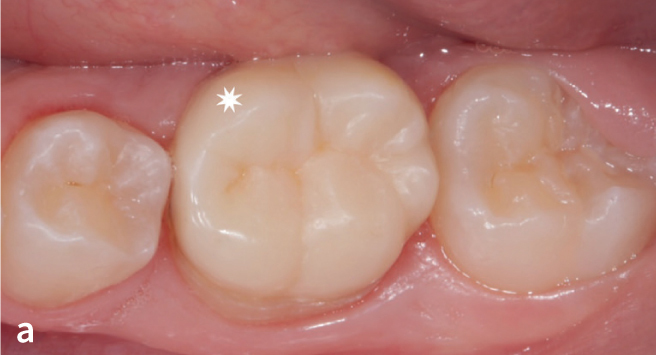
ICR on tooth 36 remained fully intact: Success (S).

**Fig 9b:**
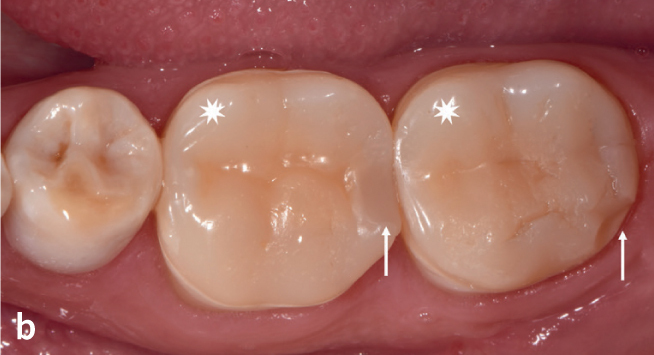
ICRs on teeth 36 and 37 required proximal shape correction (contact point creation) and repair after a chipping fracture, respectively: Survival with repair (SR).

**Fig 9c:**
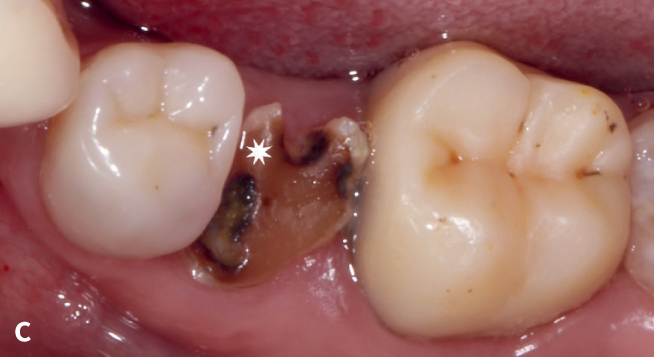
ICR on tooth 35 was lost due to secondary caries: Failure (F).

### Analysis of Periodontal Data

Overall, oral hygiene was very good in the study population (n = 34). The mean plaque score in the maxillary anterior region was 1.58 ± 0.61 (min 0.47, max 3.33, median 1.5) with a 68% CI of 1.18 to 2.12.

The results of the descriptive data analysis for PD, CAL, SBI, and TPI are presented in Table 2. There was no significant difference in the observed mean PD and CAL values between the test and CT (serial t-test; PD: p = 0.058; CAL: p = 0.15), and all values were within the physiological range. Similarly, there was no significant difference in SBI between TT and CT (p = 0.62). However, TT showed significantly higher plaque indices than CT (p = 0.001).

**Table 2:** Descriptive data analysis for PD, CAL, SBI, and TPI of TT and CT

PD (mm)	Mean	Dispersion between subjects	Dispersion within a subject	p-value
TT	1.81	0.30	0.045	0.058
CT	1.87			
CAL (mm)				
TT	1.92	0.336	0.062	0.15
CT	1.88			
SBI (0–5)				
TT	0.19	0.043	0.061	0.62
CT	0.18			
TPI (0–5)				
TT	1.89	0.351	0.179	0.0001***
CT	1.69			

n = 30 subjects, n = 52 pairs of test and control teeth. p from serial t-test, p <0.0001 is indicated with asterisks (***). Abbreviation: TT = Treated teeth, CT = Control teeth, PD = Pocket depth, CAL = Clinical attachment level, SBI = Sulcus bleeding index, TPI = Turesky Plaque Index

### Analysis of Clinical Examination Data

Of the 155 restorations included in the study, 154 were evaluated for clinical quality according to the FDI criteria (Table 3).^31^ One restoration was lost to follow-up due to failure (secondary caries) and was therefore excluded from the analysis. The clinical quality of the restorations was predominantly excellent, good, and satisfactory. In terms of esthetic, functional, and biological criteria, the restorations exhibited the typical quality issues associated with composite materials, such as a tendency to lose surface gloss, surface discoloration, and increased wear compared to dental hard tissue.

**Table 3:** FDI criteria for clinical evaluation of 154 ICRs in 34 subjects (one ICR lost to follow-up due to “failure” was excluded from the analysis)

FDI criteria		(1)	(2)	(3)	(1) – (3)	(4)	(5)	(4) – (5)
		Excellent	Good	Sufficient		Unsatisfactory	Poor	
Esthetic parameters	Surface luster	99 (64.3%)	48 (31.2%)	7 (4.5%)	154 (100%)	0 (0%)	0 (0%)	0 (0%)
	Surface staining	89 (57.8%)	56 (36.4%)	7 (4.5%)	152 (98.7%)	2 (1.3%)	0 (0%)	2 (1.3%)
	Color stability/ translucency	137 (89.0%)	13 (8.4%)	4 (2.6%)	154 (100%)	0 (0%)	0 (0%)	0 (0%)
	Anatomic form	137 (89.0%)	10 (6.5%)	5 (3.2%)	152 (98.7%)	2 (1.3%)	0 (0%)	2 (1.3%)
Functional parameters	Fracture and retention	145 (94.2%)	2 (1.30%)	4 (2.6%)	151 (97.1%)	3 (2.0%)	0 (0%)	3 (2.0%)
	Marginal adaptation	103 (66.9%)	37 (24.0%)	14 (9.1%)	154 (100%)	0 (0%)	0 (0%)	0 (0%)
	Wear	71 (46.1%)	68 (44.2%)	15 (9.7%)	154 (100%)	0 (0%)	0 (0%)	0 (0%)
	Participant’s opinion	121 (78.6%)	32 (20.8%)	1 (0.6%)	154 (100%)	0 (0%)	0 (0%)	0 (0%)
Biological parameters	Postoperative hypersensitivity	148 (96.10%)	6 (3.90%)	0 (0%)	154 (100%)	0 (0%)	0 (0%)	0 (0%)
	Caries, erosion, abfraction	140 (90.9%)	6 (3.9%)	6 (3.9%)	152 (98.7%)	2 (1.3%)	0 (0%)	2 (1.3%)
	Periodontal response	7 (4.6%)	104 (67.5%)	39 (25.3%)	150 (97.4%)	4 (2.6%)	0 (0%)	4 (2.6%)
	Integrity/cleanability	150 (97.4%)	4 (2.6%)	0 (0%)	154 (100%)	0 (0%)	0 (0%)	0 (0%)

### SEM Analysis

The results of the descriptive data analysis are presented in Table 4. The mean gap width was 135.66 ± 126.69 µm and the percentage of restorations with perfect margins was 63.85 ± 26.86%. Table 5 shows the results for the four indication groups. There were significant differences between all indication groups for the marginal quality criteria “continuous margin” and “positive step” (p <0.05). All other marginal quality criteria were not statistically different between the four indication groups (p >0.05).

**Table 4:** Descriptive analysis of SEM data for total length, mean gap width [µm] and marginal qualities “continuous margin,” “positive step,” “negative step,” “marginal gap,” “marginal fracture,” and “not applicable” [%]

	n	Mean	SD	Median	68%–CI		Min	Max
Total length [µm]	29	10608.87	4643.19	10796.02	5336.41	15957.24	2659.70	16962
Mean gap width [µm]	29	135.66	126.69	88.99	51.15	198.03	36.78	584.80
Continuous margin [%]	29	63.85	26.86	68.60	37.07	92.24	0	100
Positive step [%]	29	25.44	27.40	22.11	0	54.22	0	100
Negative step [%]	29	4.90	9.32	0	0	9.95	0	36.56
Marginal gap [%]	29	1.30	2.37	0	0	3.40	0	9.43
Marginal fracture [%]	29	4.51	6.74	1.96	0	9.53	0	27.76
Not applicable [%]	29	22.66	18.11	20.85	5.47	40.58	0	79.65

**Table 5:** Descriptive analysis of SEM data of the indication groups (I–IV) for total length, mean gap width [µm] and marginal qualities “continuous margin,” “positive step,” “negative step,” “marginal gap,” “marginal fracture,” and “not applicable” [%]

	Indication	n	Mean	SD	p_kw_
Total length [µm]	I	7	10278.64	4747.67	0.95
	II	3	10361.20	6677.84	
	III	13	11098.02	4037.74	
	IV	6	10058.17	5961.75	
Mean gap width [µm]	I	6	105.08	74.00	0.87
	II	2	77.87	15.72	
	III	10	184.31	174.75	
	IV	5	98.17	43.86	
Continuous margin [%]	I	7	72.23	14.24	0.023*
	II	3	90.37	14.41	
	III	13	48.24	24.92	
	IV	6	74.63	30.47	
Positive step [%]	I	7	20.82	19.17	0.050*
	II	3	4.95	8.58	
	III	13	38.94	31.28	
	IV	6	11.83	20.19	
Negative step [%]	I	7	1.24	2.14	0.19
	II	3	0.00	0.00	
	III	13	8.57	11.72	
	IV	6	3.69	9.03	
Marginal gap [%]	I	7	1.30	1.78	0.95
	II	3	1.30	2.25	
	III	13	1.68	3.15	
	IV	6	0.47	0.68	
Marginal fracture [%]	I	7	4.41	7.08	0.61
	II	3	3.38	3.76	
	III	13	2.58	3.32	
	IV	6	9.39	11.10	
Not applicable [%]	I	7	28.17	25.37	0.83
	II	3	16.15	9.84	
	III	13	22.47	16.12	
	IV	6	19.91	17.99	

Indication I = Persistent primary teeth / infraocclusion Indication II = Congenital structural anomalies: Amelogenesis imperfecta, dentinogenesis imperfecta Indication III = Acquired structural anomalies: Molar incisor hypomineralization Indication IV = Long-term temporary restoration due to erosion, abrasion pkw from rank variance analysis according to Kruskal and Wallis, p < 0.05 is indicated with asterisk (*).

## Discussion

This two-center observational study is the follow-up project to a short-term case series previously published by our working group.^20^ In addition to participants from our university policlinic, participants from a private practice for pediatric dentistry were also included in the present study, increasing the sample size to 34 subjects with 155 restorations and a mean observation period of 5.7 years (max. 11.8 years). In the present study, the survival and success rates for ICRs were 98.9% and 78.8%, respectively. Therefore, the ICRs evaluated in this study achieved similar or better success and survival rates than those in comparable studies,^5,13,14,20,21,56^ reflecting the advances in dentin bonding agents and composite resins over the years.

Fennis and others^21^ reported 5-year success and survival rates for small Class II indirect composite cusp restorations of 85.7% and 87%, respectively. Data by Barabanti et al^5^ showed 10-year success rates of 91% to 94% with a 100% survival rate. The discrepancy between these and our results is likely due to the fact that Barabanti’s group consistently adhered to the recommended minimum restoration thickness of 2 mm for Class I and II inlays/onlays in their study, whereas in the present study we deliberately went below the manufacturer’s recommended minimum layer thickness. The recommended minimum layer thickness for SR Adoro is 1.5 to 2.0 mm in the occlusal load-bearing area according to the user manual.^34^ Therefore, it is not surprising that our success and survival rates were somewhat lower than those reported by Barabanti et al.^5^ Another explanation may be given by the findings of Fan et al.^18^ who demonstrated that the risk of failure increased with the size of the restoration, as restorations in the present study were predominantly large but flat. Nevertheless, the data show that the ICRs studied are high-quality, minimally invasive restorations with high survival and success rates, and that they are particularly well suited for long-term temporization in children and adolescents to bridge the gap until they are old enough for definitive (all-ceramic) restorations.

The survival rate of the present ICRs after 10 years was higher than that of all-ceramic restorations by Klink and others in the treatment of non-carious defects (98.9% vs 91.4%).^35^ This can be attributed to the fact that most defects could be repaired in the present case series, whereas irreparable fractures of the restorations occurred more frequently in the other study.

The survival rate of ICRs exceeds that of stainless-steel crowns^27^ after a period of 3 years (100% vs 95%). Therefore, the research hypothesis was accepted. In the present study, almost all restorations (n = 21) experiencing adverse events such as chipping, secondary caries, decementation, and shape correction were saved by intraoral repair and maintenance, and only one restoration failure occurred during the entire observation period.

It is important to note that permanent teeth continue to elongate after all permanent teeth have erupted, also referred to as posteruptive elongation.^32,33^ In children and adolescents, especially those with a structurally compromised dentition, early crowning results in supragingival displacement of the restoration margins over time. In addition to esthetic problems, this can lead to tooth hypersensitivity and the need for subsequent retreatment. This can be avoided with our minimally invasive approach.

The microfilled composite resin used in this study showed an increased loss of surface luster over time compared to natural enamel. This resulted in increased plaque accumulation and discoloration compared to natural enamel. However, increased plaque accumulation did not lead to gingivitis in our study population. When gingivitis did occur, it was likely due to patient-related factors rather than restoration-related factors (see TPI and SBI values in test vs CT).

Surface discoloration and surface roughness were generally not an esthetic concern and were easily removed by simple polishing with aluminum oxide brushes or professional tooth cleaning. Loss of surface luster, increased plaque accumulation, and lower wear resistance^63^ are common disadvantages of composite materials, affecting both sculptable and industrial composites.^4,9,29,38^ Although ceramic materials are superior to composites in this regard, they are more brittle and prone to fracture. When used to fabricate thin restorations, they are inferior to composites in this application.^41,52^ However, newer short-term data showed promising results for ultra-thin occlusal veneers made of lithium disilicate.^53^ In this study, higher complication rates were observed with ICRs. It remains to be seen how ceramic restorations will perform in the long term when the recommended minimum layer thickness is not achieved and in the indications treated in the case series presented here.

The present study population consisted of participants from a university hospital and an independent private practice specializing in pediatric dentistry. Therefore, we believe that the data are representative. The participants’ general oral hygiene, as measured by the Turesky Plaque Index (TPI mean score 1.58 ± 0.61) score for the maxillary anterior teeth, was predominantly good.

The recall rate of 77% was relatively high, especially considering the retrospective nature of the follow-up.

The marginal quality analysis showed that 63.85% of the restorations had perfect margins. The proportion of marginal gaps or marginal defects requiring repair was low.

The mean gap width was 135.66 ± 126.69 µm (median: 89 µm), which is within the range of widths reported in other studies.^22,23,25,37,42,48^ Since the largest gap widths tended to occur in older restorations, we speculate that wear of the thin margins (eg, due to later polishing) increased the distance between the restoration margin and the preparation margin. Nevertheless, the gap was usually still completely filled with luting composite. However, these results must be interpreted with caution because only 29 of the 34 teeth had supragingival margins accessible for examination (buccal/vestibular and oral/lingual/palatal sites).

One disadvantage of this retrospective study is that there is no control group due to the retrospective design. In addition, the restorations were placed for different indications, which makes it difficult to compare them. During the statistical analysis, we performed group comparisons between subjects with MIH (indication group III) and subjects without MIH (indication groups I, II, and IV). Due to the different group sizes and small number of cases, no difference in survival and clinical quality was found between the two groups. Therefore, the results of these group comparisons have not been included in the results section. However, we believe that some findings are worth mentioning. MIH subjects had significantly more plaque (mean TPI score 2.1) than subjects of the other indication groups (mean TPI score 1.7), although the difference remained small and without pathological significance. This could be due to the significantly lower age of the MIH subjects compared to the other indication groups (see Table 1). The marginal quality in the MIH group was slightly, but still significantly, worse than that of the other indication groups. One explanation for this result could be that subjects with MIH are very young when they are treated. In addition, in many cases hypersensitivity made the treatment conditions more difficult. Based on the photographs taken of all restorations in this study, the authors can confirm that the restoration margins of all MIH participants were completely extended into sound enamel. Therefore, an insufficient adhesive bond to hypomineralized enamel as a confounding factor can be excluded.

Another limitation of the study is that ICRs have not been widely used in dentistry, probably due to their complex fabrication process. CAD/CAM technology is on the rise and will undoubtedly continue to push this particular type of restoration into the background because the CAD/CAM workflow is simply more time-efficient. However, as a university clinic with special expertise in the restorative treatment of non-caries-related tooth structure defects, we believe that individual layering is indicated in cases where thin margins are required and for anterior restorations where high esthetic expectations must be met by using a variety of materials with different translucencies.

## Conclusions

The results of this retrospective study can be summarized as follows:

- The 1-, 5-, and 10-year success rates for ICRs are 95.4%, 87.4%, and 78.8%, respectively.
- FS rates at 1, 5, and 10 years were 100.0%, 98.9%, and 98.9%, respectively. When restorations with minor defects were successfully repaired, there were no subsequent defects.
- The clinical quality of the restorations was predominantly excellent to good. The restored teeth showed a slight tendency toward loss of surface gloss, surface discoloration, wear, and plaque accumulation.
- ICRs had no negative effect on periodontal health despite increased plaque accumulation.

We conclude that ICRs are suitable for minimally invasive restoration of large tooth structure defects in the developing dentition of children and adolescents and for long-term temporary restoration of the adult dentition.
